# Pathomechanisms of Autoimmune Based Testicular Inflammation

**DOI:** 10.3389/fimmu.2020.583135

**Published:** 2020-09-25

**Authors:** Livia Lustig, Vanesa A. Guazzone, María S. Theas, Christiane Pleuger, Patricia Jacobo, Cecilia V. Pérez, Andreas Meinhardt, Monika Fijak

**Affiliations:** ^1^Departamento de Biología Celular e Histología/Unidad Académica II, Facultad de Medicina, Universidad de Buenos Aires (UBA), Buenos Aires, Argentina; ^2^Instituto de Investigaciones Biomédicas (INBIOMED), Consejo Nacional de Investigaciones Científicas y Tècnicas (CONICET), Universidad de Buenos Aires (UBA), Buenos Aires, Argentina; ^3^Department of Anatomy and Cell Biology, Justus-Liebig University Giessen, Giessen, Germany; ^4^Hessian Centre of Reproductive Medicine, Justus-Liebig University Giessen, Giessen, Germany

**Keywords:** testicular inflammation, autoimmunity, experimental autoimmune orchitis (EAO), infertility, testis immunoregulation

## Abstract

Infection and inflammation of the male reproductive tract are relevant causes of infertility. Inflammatory damage occurs in the special immunosuppressive microenvironment of the testis, a hallmark termed testicular immune privilege, which allows tolerance to neo-antigens from developing germ cells appearing at puberty, long after the establishment of systemic immune tolerance. Experimental autoimmune orchitis (EAO) is a well-established rodent model of chronic testicular inflammation and organ specific autoimmunity that offers a valuable *in vivo* tool to investigate the pathological and molecular mechanisms leading to the breakdown of the testicular immune privilege. The disease is characterized by the infiltration of the interstitium by immune cells (mainly macrophages, dendritic cells, and T cells), formation of autoantibodies against testicular antigens, production of pro-inflammatory mediators such as NO, MCP1, TNFα, IL6, or activins and dysregulation of steroidogenesis with reduced levels of serum testosterone. EAO leads to sloughing of germ cells, atrophic seminiferous tubules and fibrotic remodeling, parameters all found similarly to changes in human biopsies from infertile patients with inflammatory infiltrates. Interestingly, testosterone supplementation during the course of EAO leads to expansion of the regulatory T cell population and inhibition of disease development. Knowledge of EAO pathogenesis aims to contribute to a better understanding of human testicular autoimmune disease as an essential prerequisite for improved diagnosis and treatment.

## Introduction

Infection and inflammation of the male reproductive tract are relevant causes of infertility with a prevalence of 6–10% (1, 2). Bacterial infections eliciting epididymo-orchitis are either sexually transmitted or originate from urinary infections often resulting from ascending canalicular infections of the male excurrent ducts. Also, a number of systemically transmitted viruses (mumps virus, HIV and ZIKV virus, among others) are able to induce orchitis. Inflammation of the male reproductive tract is associated not only with infections but also with aging and diseases that damage germ cells (GC) including testis cancer, obesity, cryptorchidism and systemic autoimmune diseases, as well as trauma and toxic agents (3–7).

Autoimmune infertility has long been postulated as one of the causes of infertility, even though a well-defined entity has not yet been established. However, it is relevant that focal lymphocytic infiltrations have been detected in 25–30% of testicular biopsies from infertile patients (8, 9). Anti-sperm antibodies (ASA) have been mainly detected in patients with obstructive azoospermia (10). However, there is so far no close association between ASA and male genital tract inflammation (11, 12) except for some reports associating ASA with idiopathic granulomatous orchitis (13, 14) and a history of epididymitis/orchitis (15). Our own studies reported significantly elevated titers of autoantibodies against disulphide isomerase family A, member 3 (ER-60) in sera from infertile azoospermic patients with histologically confirmed low-grade testicular inflammation (16). Further, the major evidence for an autoimmune basis of human orchitis comes from patients with autoimmune polyendocrine syndrome APS-type 1 caused by AIRE gene mutation which induces testis impairment and antibodies to Leydig cell antigens (17).

Experimental autoimmune orchitis (EAO) is a well-established rodent model of organ specific autoimmunity that provides a very valuable *in vivo* tool to investigate pathological, immune and molecular mechanisms involved in chronic testicular inflammation.

## Experimental Models of Autoimmune Orchitis

Research on spontaneous autoimmune orchitis in mink (18, 19) and rodents after vasectomy (20), thymectomy (21, 22), or genetic manipulation (23) have constructed a body of evidence for better understanding of systemic and peripheral tolerance. Studies in classical rodent EAO models induced by antigen immunization have also clarified pathological mechanisms of autoimmune-based testicular inflammation [reviewed in (8, 24)].

The first report on a classical EAO model was published by Voisin et al. (25) who injected testicular tissue and adjuvants into guinea pigs. However, it was Freund et al. (26) who clarified the organ and species specificity of the model. In a murine model, EAO susceptibility depends of genetic background of each strain (27). Further progress on the pathogenesis of EAO has come from mouse and rat models, initiated by Tung and colleagues (28) and Doncel et al. (29), respectively. A description of the histopathology, mechanisms of disease initiation and testicular inflammation are discussed below.

Current rat and mouse EAO models utilizing testicular homogenate in complete Freund's adjuvant plus *pertussis* toxin (8), vasectomized mouse model (20), and mouse EAO model without adjuvants (30) have provided novel insights into orchitogenic antigens. Most of these antigens, identified by sera obtained from animals with EAO, are not testis-specific except zonadhesin or outer dense fiber major protein 2 (30–32).

## Immune Privilege of the Testis

Testicular homeostasis that protects GC from immune attack is known to be maintained by structural components such as the blood-testis barrier (BTB) and systemic and local tolerance mechanisms. In contrast with the previous testis antigen sequestration paradigm, some meiotic germ cell antigens, located in the adluminal compartment of the seminiferous tubules (ST) behind the BTB, are continuously released into the interstitial space despite an intact BTB. Systemic tolerance involving antigen-specific regulatory T cells (Tregs) is maintained in peripheral lymphoid organs by continuously egressing germ cell antigens via transcytosis in Sertoli cells (33). Originally, the testis was defined as an immune-privileged site since it was demonstrated that foreign-tissue grafts placed within the testis are tolerated and survive for several days longer than when these grafts are implanted in conventional body sites (34). Currently, testicular immune privilege is understood as the coordinated regulation of immunologic components to protect GC, including active processes associated with Sertoli cells, peritubular cells, Leydig cells, tolerogenic antigen-presenting cells, T cells and the production of immune-regulatory factors such as TGFβ, IL10, and activin [reviewed in (35–37)]. Several reviews suggest galectin-1 (*Lgals1*) as a putative candidate involved in the maintenance of testis immune privilege, mainly based on its expression by Sertoli cells (38–40). However, a significant reduction in the incidence and severity of EAO was observed in Lgals1^−/−^ deficient vs. wild-type mice (41) adding a note of caution to this discussion. Indoleamine-2,3-dioxygenase (IDO) expression in porcine Sertoli cells and the ability of these cells to restore immune tolerance in NOD mice (42) point to a role of IDO in testis immune privilege. Current findings from functional *in vivo* experimental studies confirm that tryptophan metabolism modulates inflammatory immune response to spermatic antigens (43).

The BTB is formed by cell junctions of adjacent Sertoli cells at the base of the ST. It is constituted by multiple cell junction types including tight junctions, basal ectoplasmic specializations, gap junctions and desmosome-like junctions. Various integral tight junction proteins have been described between adjacent Sertoli cells with occludin and claudin 11 being the most important for barrier integrity (44). These proteins link to the actin cytoskeleton via cytoplasmic plaque proteins including zonula occludens-1,−2, and−3, and provide links to other junctional types (gap-, adherens-) in the BTB (45). Ectoplasmic specialization-mediated adhesion is largely constituted by the cadherin-catenin multifunctional complex (46). Gap junctions are cell-cell channels that allow diffusion of metabolites, second messengers, ions, and other molecules smaller than 1 kDa, being Cx43 the dominant gap junction protein within the ST (47). Testosterone, nitric oxide (NO), cytokines and growth factors regulate the stability or localization of proteins at the BTB (48–50). The BTB is a dynamic ultrastructure that transiently “opens” and “closes” during the movement of preleptotene/leptotene spermatocytes to the adluminal compartment without causing failure of BTB function. As leptotene spermatocytes transit toward the tubule lumen, junction disassembly ahead of spermatocytes is coordinated by junction assembly behind these GC so that the two spermatogenic events are synchronized (46). Moreover, the presence of specific transporters located along the basolateral membrane of Sertoli cells that allow the passage of selective molecules while restricting the entry of others, makes the BTB a physiological barrier crucial for the development and maturation of GC.

## Histopathology and Mechanisms of Testicular Inflammation in Rodent EAO Model

**Figure 1:**
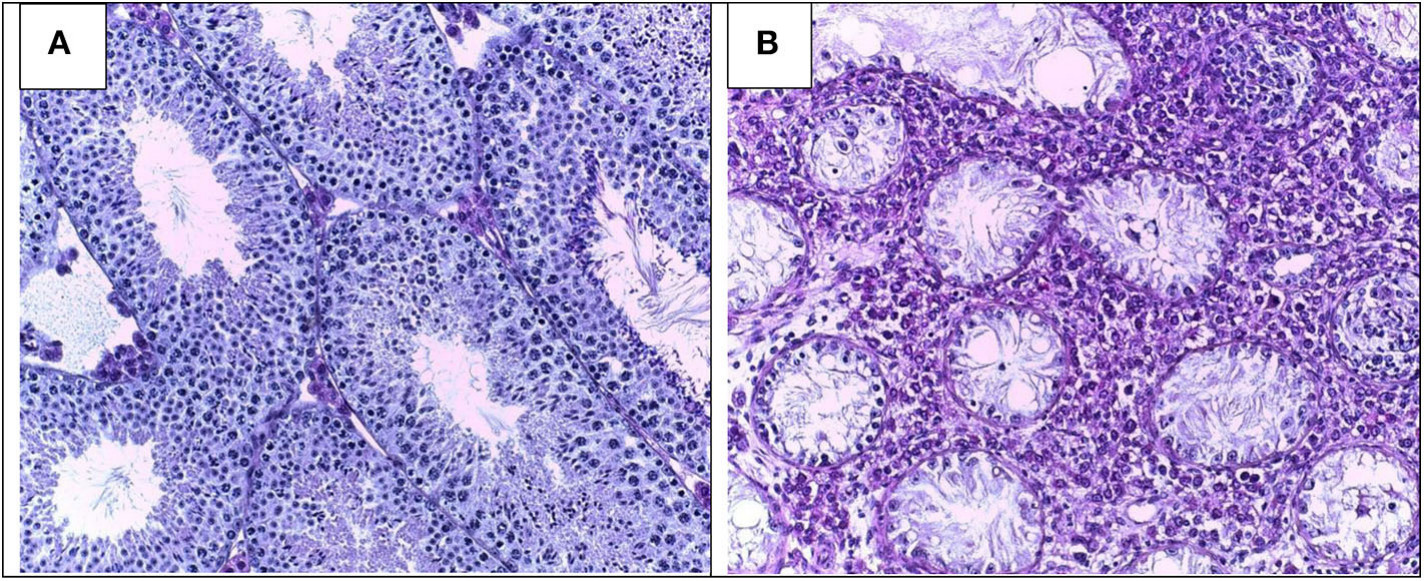
Testicular histology in normal **(A)** and severe EAO **(B)** mouse testis. Typical histopathological changes include infiltration of the interstitium by immune cells, sloughing of germ cells leading to aspermatogenesis, vacuolization of Sertoli cells cytoplasm, thickening of lamina propria, extensive necrosis, and fibrosis of seminiferous tubules (magnification × 200).

**Figure 2:**
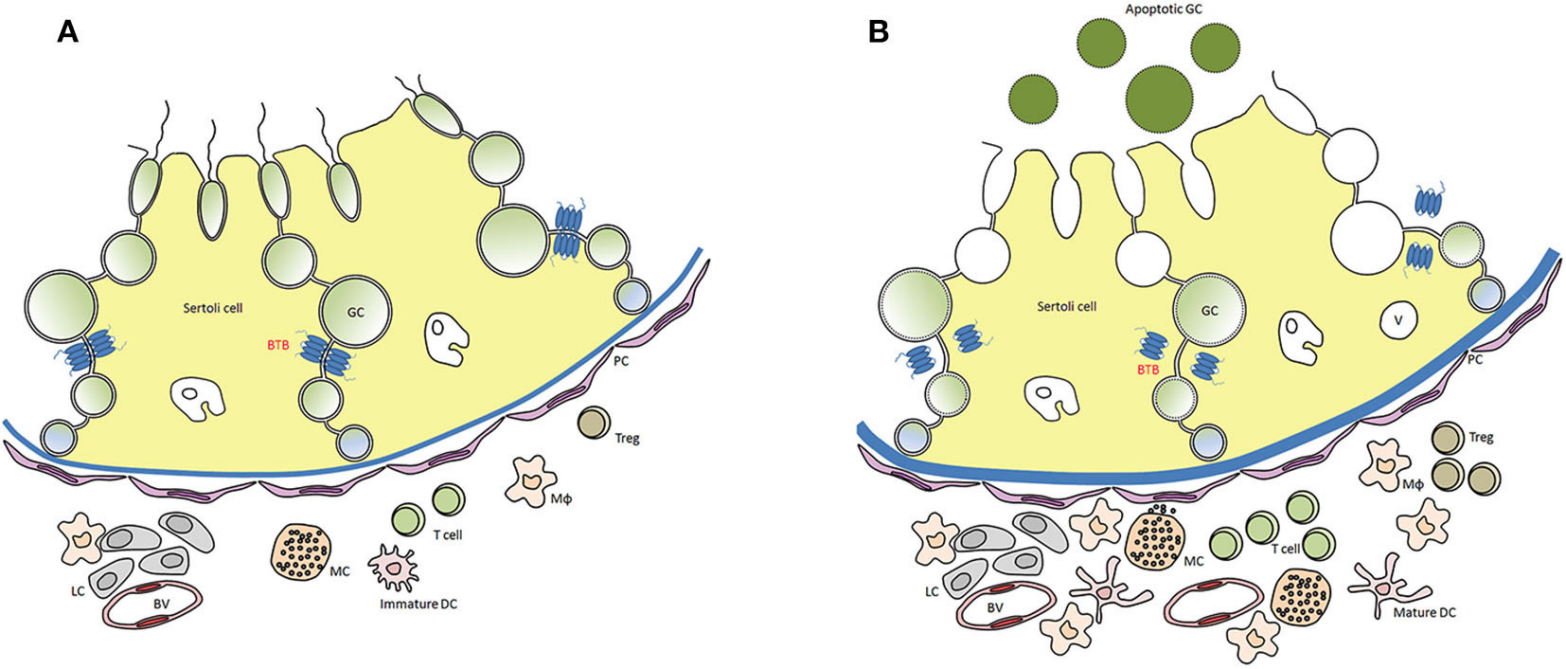
Distribution of immune cells in a rodent testis section under normal **(A)** and inflammatory conditions (EAO) **(B)**. In EAO, dendritic cells (DC), macrophages (MΦ), T cells, regulatory T cells (Treg), and mast cells (MC) are increased in number and distributed in the interstitium, mainly in the peritubular area of damaged seminiferous tubules (ST). Some MC are located in a close proximity to peritubular cells (PC). Impairment of blood testis barrier (BTB) and disturbances of spermatogenesis (presence of apoptotic germ cells in the ST lumen) are illustrated; BV, blood vessel; v, vacuole in Sertoli cell cytoplasm [modified from (51)].

Testis histopathology in EAO is characterized by an increase in the number of dendritic cells (DC), macrophages (Mϕ), mast cells and T lymphocytes (L) distributed in the interstitium close to ST, exhibiting different degrees of GC sloughing (mainly spermatids and spermatocytes). Germ cell apoptosis and multinucleated spermatids in the lumen of ST as well as vacuolization in the cytoplasm of Sertoli cell are frequently observed. Damage of ST is initially focal, followed by development of severe orchitis showing aspermatogenesis and fibrosis of the wall of most ST (Figure 1). L and Mϕ distributed in the interstitium might enter the ST in mice as well as in humans in contrast to rats. Large granulomae are frequently observed. Hyperplasia and hypertrophy of Leydig cells and an increased number of small blood vessels are also detected (Figure 2).

### Blood Testis Barrier

During testicular inflammation BTB integrity is impaired—denoted by increased permeability to tracers (52, 53). Concomitantly, changes in expression of cell junction adhesion molecules were detected. A decrease in occludin and Cx43 expression and an increase in the expression of N-cadherin and α-catenin were observed in testis of rats with EAO (53, 54).

Increased levels of inflammatory cytokines in the EAO testis alter the normal function of the BTB. Local administration of IL17A into the rat testis increases BTB permeability by reducing occludin expression and delocalization of claudin-11 (55). IL6 impairs the Sertoli cell tight junction barrier in normal rats by perturbing the MAPK14 signaling pathway and inhibiting BTB-constituent protein degradation (54, 56). TNFα administered locally to adult rat testis inhibits the steady-state protein levels of occludin, ZO-1, and N-cadherin altering the BTB function (57).

### Dendritic Cells

The maturation state of DC cells is regarded as a control point for the induction of peripheral tolerance or autoimmunity. Purified DC from EAO rat testes demonstrated significantly upregulated expression of the chemokine receptor CCR7, which is responsible for the migration of DC to the draining lymph nodes (58). Moreover, the expression of IL10 and IL12p35 transcripts were detectable only in DC from inflamed testes, pointing to a mature immunogenic state before imminent migration to the lymph nodes. DC in draining lymph nodes from rats with EAO are mature, present antigens to T cells, and stimulate an autoimmune response against testicular antigens, thereby causing immunological disturbances of the testis (59).

### Macrophages

A large population of resident Mϕ subsets in the interstitium of normal testis is in close association with Leydig cells (60). Testicular Mϕ modulate spermatogenesis, steroidogenesis and also BTB permeability in the normal testis (61). Once testicular immune privilege is disrupted by immunization with spermatic antigens, the number of Mϕ progressively increases in the testicular interstitium. Chemokines, mainly MCP1, acting on endothelial cells, facilitate infiltration into the testis of CD68^+^ CD163^−^ monocytes from circulation, expressing the MCP1 receptor CCR2 (62, 63). Most of these infiltrating Mϕ express MHCII molecules and secrete pro-inflammatory mediators, mainly TNFα, IL6, and NO involved in BTB impairment, lymphocyte infiltration, and germ cell apoptosis (53, 64–66). Depletion of Mϕ by *in vivo* administration of liposomes containing clodronate significantly reduced the incidence and severity of EAO, thereby highlighting the requirement of these cells for disease induction (63).

### T Cells

From the early focal EAO stage onwards, a large increase in the number of CD4^+^ and CD8^+^ T cells occurs in the interstitium in association with increasing damage of ST (67). Interestingly, in a mouse EAO testis, a population of double positive CD4^+^CD8^+^ T cells was detected (68). However, the function of these cells in the periphery is not very well-investigated. TNFα and IFN⋎-producing Th1 cells and IL17-producing Th17 cells govern the early stage of EAO while CD8^+^ T cells (TNFα^+^, IFNγ^+^, and IL17^+^) lead the disease to its chronic severe stage (69). Besides being the main source of Th1 and Th17 cytokines in the chronically inflamed testis, CD8^+^ cells are the main subset expressing the CD25 activation marker (67). Together with the influx of auto-pathogenic T cells, CD4^+^ and CD8^+^ cells with regulatory phenotype (Tregs) also accumulate in EAO testis and in the lymph nodes draining the testis (TLN) (67, 70). These subsets show dynamic behavior during EAO progression; interstitial CD4^+^Foxp3^+^ Tregs reach a maximum number at the early stage of EAO and then decline at the chronic stage. However, the number of CD8^+^Foxp3^+^ subset that increases to a lesser extent than their CD4^+^ Foxp3^+^ counterparts during EAO onset remains stable throughout disease. The regulatory function of this subset was not evaluated. Most CD4+ Foxp3+ Tregs from TLN display an antigen-experienced phenotype and also express TGF-β. *In vitro* suppression studies showed that CD4^+^Foxp3^+^ Tregs derived from EAO TLN suppress T cell proliferation more efficiently than their counterparts derived from normal TLN, suggesting that EAO Tregs are over-activated by inflammation (70). However, Tregs present in the testicular inflammatory microenvironment fail to counteract deleterious autoimmune effects on GC; consequently, tissue damage progresses (71).

### Germ Cells

In EAO testis, GC death occurs through apoptotic mechanisms involving FasL-Fas, TNFα-TNFR, IL6-IL6R, and NO-NOS systems, via autocrine or paracrine pathways. The number of apoptotic GC sloughed from the seminiferous epithelium expressing Fas, TNFR, or IL6R increases with progression of testicular damage (66, 72, 73). With the influx of T cells expressing FasL, the content of the soluble form of FasL increases in the interstitial fluid. This factor is capable of crossing the altered BTB in inflammation, and triggers Fas-sensitive germ cell apoptosis (74). TNFα produced in large quantities by resident Mϕ, infiltrating monocytes, and also by T cells induces apoptosis of spermatocytes and spermatids expressing TNFR1 (66, 72, 74). IL6 secreted by infiltrating Mϕ, Leydig, and peritubular cells activates executioner caspase-3, resulting in apoptosis of IL6R^+^ GC (73).

An oxidative microenvironment is generated in EAO testis by high levels of NO, produced mainly by both resident and infiltrating Mϕ (65). NO reaches ST and induces basal germ cell apoptosis by activating the mitochondrial pathway. Spermatogonia are likely sensitive to oxidative stress generated by NO since DETA-NO, a compound that releases NO, induces cell cycle arrest and apoptosis in GC-1 cell line (75).

Apoptosis in EAO testis occurs via a cytokine-dependent amplification loop resulting from the activation of death receptors and oxidative damage of GC. This phenomenon is sustained by the ongoing influx of immune cells into the testicular interstitium.

## Fibrotic Response—Involvement of Activin A

In severe EAO fibrotic remodeling is a hallmark of the disease. Fibrotic changes are initiated around the ST and show excessive production and deposition of extracellular matrix proteins such as fibronectin and collagens and a thickening of the lamina propria. The fibrotic alterations are accompanied by changes in the morphological appearance and thickening of the α-smooth muscle actin layer in the peritubular cells, belonging to the population of myofibroblasts in the testis (68, 76). Expression of fibronectin correlates positively with the disease damage score and fibrosis. Moreover, there is strong correlation between activin A concentrations and fibrotic damage in EAO testis (76). These changes show—also in human biopsies from patients with Sertoli cell only syndrom—a clear relationship between activin A expression—and lymphocytic infiltrates (76). Stimulation of peritubular cells by activin A increased levels of fibronectin and collagen I and IV implicating activin A as an important mediator of fibrotic remodeling during testicular inflammation (76). This underlines a function for activin A beside its classical endocrine function in inducing follicle stimulating hormone (FSH) secretion from the pituitary but increasingly as an important regulator of inflammation and fibrosis in many organs (77). Activin A is broadly expressed in testicular cells and stimulation of cultured Sertoli cells with TNFα leads to elevated expression of activin A (76). However, elevating circulating levels of follistatin, an endogenous antagonist of the activins prior to EAO induction was not sufficient to fully inhibit the disease development, although the severity of the disease and the extent of the fibrotic damage was reduced (52).

## Hormonal Regulation During Inflammatory Phase of the Disease

The hormonal status of EAO animals shows disturbances of the hypothalamic-testicular axis at several levels. In EAO rats the levels of FSH are increased while the concentration of testosterone in serum is significantly downregulated (78–80). In contrast, the levels of intratesticular testosterone were either upregulated or unchanged, depending on the scientific report (78–80). Earlier *in vitro* experiments demonstrated that basal and human chorionic gonadotropin (hCG) stimulated testosterone production were significantly elevated in EAO testis (81). Moreover, Leydig cells from EAO testis *in vitro* showed an increased basal and hCG stimulated testosterone production compared to cells from control animals. Stimulation with TNFα inhibited this effect (72). Interestingly, testosterone substitution of EAO rats demonstrated a reduction of macrophage accumulation and CD4+ T cell influx at the testicular level, while the numbers of regulatory CD4+CD25+Foxp3+ T cells were increased. Testosterone treatment induced a strong increase in the number of regulatory T cells *in vivo* and *in vitro* (80, 82). Of note, androgens through androgen receptor modulate expression of Foxp3 (83) (Table 1).

**Table 1:** Pathological events leading to development of experimental autoimmune orchitis (EAO).

Structural and functional changes in Sertoli cells	Impairment of BTB structure and function by the action of pro-inflammatory cytokines mainly secreted by immune cells Vacuolization of Sertoli cell cytoplasm
Immunopathology	Testicular dendritic cells (DC) become mature, migrate to the testis-draining lymph nodes (TLN) and activate T cells
	Inflammatory macrophages (MΦ), DC, effector T lymphocytes (Th1, Th17, and CD8^+^) and mast cells (MC), infiltrate the testis CD4^+^ Foxp3^+^ regulatory T lymphocytes (Tregs) actively accumulate within the testis and TLN Small vessels increase in number and chemokines enhance immune cell infiltration
	Tregs present in the testis fail to counterbalance immunoreactions that cause deleterious effects on germ cells (GC)
	Antigens released from damaged seminiferous tubules (ST) amplify the autoimmune response leading to continuous antigen presentation to T lymphocytes in TLN (chronification of orchitis)
Disturbances of spermatogenesis	GC apoptosis and sloughing in the tubular lumen mainly induced by the action of TNFα, IL6, NO, and Fas ligand
Hormonal changes	Impairment of androgen production
Chronic phase of disease	Aspermatogenesis, ST atrophy, fibrosis and thickening of ST wall Infertility

## Involvement of the Epididymis

In EAO mice, the epididymis underwent a region-specific immune response positively correlating to the severity of orchitis (28, 84). Similar to the observed differential immune responsiveness in a model of acute bacterial epididymitis (85, 86), the distally located cauda epididymidis and vas deferens show a severe immune reaction in EAO mice characterized by an upregulated expression of cytokines and immunomodulatory factors (*Tgfb1, Ccl2, Il1b, Il10, Tnf, Foxp3, Ido1*), immune cell infiltration, fibrosis and epithelial damage resulting in a loss of tissue integrity and subsequent aggregation of displaced spermatozoa within the interstitium (84). The proximal regions (initial segment and caput), in contrast, do not reveal histopathological alterations or an upregulated expression of cytokines, although these regions are more densely vascularized and harbor a high number of resident immune cells (87, 88). It needs to be noted though that data on epididymal reactions in EAO models are very scarce and thus the underlying molecular pathomechanisms remain unknown.

## Discussion

In summary, rodent models of EAO offer a valuable tool to discover the decisive mechanisms for the development of autoimmune-based epididymo-testicular inflammation. However, caution is necessary in extrapolating the data from rodent models to human due to several differences at the immunological and cellular level. Silent asymptomatic testicular inflammation in human is rather difficult to diagnose and treatment hampered due to missing non-invasive diagnostic tools. Possible therapeutical interventions and the development of new non-invasive diagnostic tools, such as serum assays may offer potential developments in the field gathered from the animal model. Several previous studies were already successfully dealing with the therapeutic inhibition of EAO by using e.g., depletion of Mϕ (63), blockade of pro-inflammatory mediators (41, 52, 64, 89) or administration of testosterone (80). Supplementation of reduced testosterone levels in EAO rats led to inhibition of disease development and suggests testosterone as an immunoregulatory and immunosuppressive factor during testicular inflammation (80). Further studies are necessary to define the biomarkers and possible targets of autoimmune-based testicular inflammation.

## Main Outstanding Questions in the Field

To further our understanding of the immunopathology of autoimmune based impairment of fertility, the following aspects would warrant attention in future research: (a) the identification of molecular mechanism that trigger the autoimmune attack in the human testis without obvious presence of pathogens, (b) the evaluation of specific genetic predisposition in humans responsible for susceptibility to autoimmune diseases of the gonads, (c) elucidation of the common features of testis autoimmunity with autoimmune diseases of other organs, mainly in relation to Tregs behavior, and (d) the role of specific testicular somatic cells beside immune cells in the development of the disease.

## Author Contributions

LL, VG, MT, CP, PJ, CP, AM, and MF performed literature research and wrote the manuscript together. All authors reviewed and approved the final version of the manuscript.

## Conflict of Interest

The authors declare that the research was conducted in the absence of any commercial or financial relationships that could be construed as a potential conflict of interest.
